# Delayed auditory feedback increases speech production variability in typically fluent adults but has the opposite effect in stuttering adults

**DOI:** 10.3389/fnhum.2025.1628114

**Published:** 2025-08-29

**Authors:** Ayoub Daliri, Shogo Honda, Ludo Max

**Affiliations:** ^1^College of Health Solutions, Arizona State University, Tempe, AZ, United States; ^2^Department of Speech and Hearing Sciences, University of Washington, Seattle, WA, United States

**Keywords:** stuttering, variability, delayed auditory feedback, feedforwad control, speech

## Abstract

**Purpose:**

Extensive evidence indicates that auditory-motor integration during speech production is inefficient in individuals who stutter and that speaking with delayed auditory feedback (DAF) increases speech fluency in this population. However, the exact *mechanisms* underlying these auditory-motor integration difficulties and the DAF-induced fluency enhancement remain unknown. Here, we examined (1) whether stuttering is associated with increased variability in the feedforward component of sensorimotor control and (2) whether speaking with DAF reduces this variability in speech movement planning in adults who stutter.

**Method:**

We extracted acoustic measures from audio recordings made during a prior study that included 12 right-handed adults who stutter and 12 age-, sex-, and handedness-matched nonstuttering adults. Participants produced front vowels in the context of monosyllabic consonant-vowel-consonant words in conditions where they spoke with either normal auditory feedback (NAF) or 100-ms DAF. For each participant in each condition, we calculated trial-to-trial formant variability to assess feedforward processes.

**Results:**

Relative to nonstuttering adults, stuttering adults generally showed greater trial-to-trial formant variability. DAF had a differential influence on trial-to-trial formant variability, increasing it for non-stuttering adults but decreasing it for stuttering adults.

**Conclusion:**

These findings suggest that stuttering adults generated more variable motor commands than nonstuttering adults when speaking with NAF, but that speaking with DAF decreased variability in the stuttering group, even though it increased variability in the nonstuttering group. One possible interpretation of these findings is that stuttering modulates the sensorimotor system’s processing of auditory errors, specifically influencing how significantly these errors are weighted when the sensorimotor system determines its responses to the errors.

## Delayed auditory feedback increases speech production variability in typically fluent adults but has the opposite effect in stuttering adults

Stuttering is a speech production disorder that impairs speech fluency in approximately 1% of adults (Gattie et al., 2024a, 2024b). Accumulating neural and behavioral evidence suggests that stuttering is linked to inefficiencies in neural processes involved in speech movement planning and production (Bradshaw et al., 2021; Max and Daliri, 2019; Neef and Chang, 2024). For example, functional neuroimaging studies of stuttering have consistently reported abnormalities in activities of motor and auditory regions involved in speech planning and production (Chang and Guenther, 2020; Chang et al., 2019; Etchell et al., 2018). Supporting evidence from structural neuroimaging studies indicates that stuttering is also associated with abnormalities in structural connectivity between motor and auditory regions (e.g., Neef et al., 2015). Based on such evidence, many theories of stuttering posit that deficient or inefficient auditory-motor integration may lead to breakdowns in speech fluency of individuals who stutter (Bloodstein and Ratner, 2008). Consistent with this suggestion, fluency-enhancing conditions that influence auditory-motor processes, such as speaking with delayed auditory feedback (DAF), reduce speech disfluency and normalize brain activity in auditory-motor regions of individuals who stutter (Budde et al., 2014; Daliri and Max, 2018; Korzeczek et al., 2021; Nil et al., 2008; Sakai et al., 2009; Toyomura et al., 2015). Notably, whereas shorter auditory delays (less than 100 ms) have been reported to enhance speech fluency in individuals who stutter, longer delays (exceeding 200 ms) are often associated with increased disfluency (for a review, see Lincoln et al., 2006). Additionally, speaking with DAF increases speech disfluency for individuals who do not stutter (Soderberg, 1969; Stuart and Kalinowski, 2015; Yates, 1963).

Although theoretical and empirical evidence suggest that auditory-motor processes are inefficient in individuals who stutter, the specific *mechanisms* underlying these auditory-motor challenges and how DAF improves speech fluency remain to be fully elucidated. However, numerous previous studies on the nature and extent of inefficiencies in the auditory-motor processes of individuals who stutter have offered valuable insights (Chow et al., 2023; Civier et al., 2010; Daliri et al., 2017; Daliri and Max, 2018; Kim et al., 2020; Rowe et al., 2024). One theoretical perspective suggests that stuttering may be associated specifically with inefficiencies in feedforward processes—processes involved in preparing and optimizing motor commands (Max, 2004; Max et al., 2004). In several empirical studies, we measured brain responses to auditory stimuli presented immediately before speech onset (pre-speech auditory modulation, PSAM) as a neural signature of predictive processes occurring during speech movement planning (Daliri and Max, 2015a,b, 2018). Most recently, we also investigated the relationship between PSAM and trial-to-trial variability in acoustic speech output (formant frequencies) as a behavioral measure of feedforward processes (Li et al., 2024). For this purpose, we calculated formant variability (i.e., the distance between formants in a given trial and the median formant values across all trials) based on the early portion of speech when the brain has limited access to the auditory feedback from the ongoing production; thus, the production is primarily influenced by feedforward processes—and not by feedback processes that rely on feedback to correct the current production during the production (within-trial feedback-driven corrections) (Daliri, 2021; Guenther, 2016; Kearney et al., 2020; Merrikhi et al., 2025). We showed that, across speakers, less PSAM was associated with greater trial-to-trial variability. This finding suggests that, contrary to our previous hypothesis that PSAM reflects a fine-tuning of the auditory feedback control system (Max and Daliri, 2019), PSAM may instead reflect processes involved in optimizing feedforward motor commands (Li et al., 2024). Importantly, PSAM has been shown to be limited in stuttering individuals (Daliri and Max, 2015a,b, 2018; Mock et al., 2015), but speaking with DAF (a fluency-inducing condition) enhanced its manifestation in this group and eliminated the between-group difference with fluent speakers (Daliri and Max, 2018). Together, these findings suggest that stuttering may be associated with significant issues in feedforward motor command generation, and that DAF may have modulating effects that mitigate these problems.

Considering trial-to-trial variability of speech as a behavioral correlate of feedforward processes, one could predict that inefficiencies in the feedforward processes of individuals who stutter would manifest as increased trial-to-trial variability in their speech. An extensive set of data from multiple studies does indeed confirm that individuals who stutter exhibit greater trial-to-trial movement variability during speech (Chon et al., 2021; Jackson et al., 2016; Jäncke, 1994; Janssen et al., 1983; Kalveram and Jäncke, 1989; Loucks et al., 2022; Smith and Kleinow, 2000; Usler and Walsh, 2018; Walsh et al., 2015; Wieneke et al., 2001; Wiltshire et al., 2021). These studies have examined the similarity and repeatability of movement trajectories of orofacial structures (e.g., lips) or the acoustic speech output (e.g., voice onset time, vowel duration) over repeated production of utterances to estimate speech movement variability. However, these studies did not dissociate the contributions of the feedforward and feedback processes to the observed variability. The early parts of speech segments are predominantly shaped by feedforward processes, but the later parts reflect the combined influence of both feedforward and feedback processes (Daliri, 2021; Guenther, 2016; Kearney et al., 2020).

Therefore, the first goal of the present study was to investigate whether the speech of individuals who stutter is characterized by greater trial-to-trial formant variability, thereby using formant variability as a proxy for the efficiency of feedforward processes. Additionally, because DAF appears to play a critical role in augmenting feedforward processes in stuttering individuals, the second goal of this study was to determine whether DAF leads to a reduction in trial-to-trial formant variability in stuttering individuals. Adults who stutter (AWS) and adults who do not stutter (ANS) produced front vowels in the context of monosyllabic consonant-vowel-consonant words (a) when speaking under normal auditory feedback (NAF) and (b) when speaking under a 100-ms DAF. We hypothesized that (a) if stuttering is associated with inefficiencies in feedforward processes, AWS would have higher trial-to-trial formant variability when speaking under NAF, and (b) if DAF improves the feedforward processes of AWS, it would lead to a reduction in their trial-to-trial formant variability.

## Method

### Participants

All acoustic measures were made from audio files recorded (but not analyzed) for a previous electroencephalography experiment (Daliri and Max, 2018). This data set included 12 right-handed stuttering adults (2 females; M_age_ = 29 years, SD_age_ = 8 years) and 12 age- (±3 years), sex-, and handedness-matched nonstuttering adults (2 females; age: M_age_ = 28 years, SD_age_ = 8 years). For both stuttering and nonstuttering participants, we used the following inclusion criteria: (1) being a native speaker of American English, (2) having a binaural pure-tone hearing threshold less than 20 dB HL at octave frequencies of 250 Hz to 8,000 Hz, (3) having no history of psychological, neurological, or communication disorders (other than developmental stuttering for the stuttering participants), and (4) not taking medications that influence the sensorimotor system. We used the following inclusion criteria for the stuttering participants: (1) confirmation of the diagnosis of stuttering by one of the first two authors of this study and (2) self-reported stuttering onset during childhood (<8 years of age). We used the Stuttering Severity Instrument—Fourth Edition (Riley, 2008) to assess the stuttering severity of the stuttering participants, revealing a range from very mild to very severe. The severity of stuttering among participants was distributed as follows: four individuals had very mild stuttering, two had mild stuttering, five had moderate stuttering, and one had very severe stuttering. The University of Washington’s Institutional Review Board approved all study protocols, and all participants signed a written consent form before the experimental session.

### Experimental design

Participants sat in front of a computer monitor inside a sound-attenuating booth. A dynamic microphone (SM58, Shure Incorporated, Niles, IL) was placed approximately 15 cm in front of the participant’s mouth to collect speech signals. The microphone signal was amplified (DPS II, ART ProAudio, Niagara Falls, NY) and transmitted to an external speech processing system (VoiceOne, TC-Helicon, Victoria, BC, Canada). The output of the speech processing system was amplified (S-phone, Samson Technologies Corp., Syosset, NY) and played back to participants via insert earphones (ER-3A, Etymotic Research Inc., Grove Village, IL). Before the experiment, we adjusted the microphone and earphone amplifiers such that the signals at the earphones were 5 dB SPL higher than the microphone signal (Chao et al., 2019; Daliri and Dittman, 2019; Daliri and Max, 2018). We used a custom-written MATLAB script (The MathWorks, Inc., Natick, MA) to record the microphone signal and the earphone signals on two separate channels at a sampling frequency of 44,100 Hz.

The experiment consisted of two conditions: speaking under normal auditory feedback (NAF) and speaking under delayed auditory feedback (DAF). Participants completed three blocks of each condition in a random order (a total of 96 trials in each condition). The overall designs of the two conditions were similar. In each trial, a target word would appear on the monitor, and participants would produce the word aloud. Participants were instructed to produce the words as they would normally produce them. In the NAF condition, participants heard their normal auditory feedback through the insert earphones while speaking. In the DAF condition, participants heard their auditory feedback through the insert earphones with a 100-ms delay. It should be noted that in both NAF and DAF conditions, participants’ speech was processed by the external speech processor (VoiceOne, TC-Helicon). This processor uses a proprietary algorithm to alter the timing of input signals. Due to the processor’s inherent delay of ~10 ms (Kim et al., 2020), participants experienced this minimal delay in the NAF condition. We confirmed the delay for both NAF (i.e., ~10 ms) and DAF (i.e., 100 ms; ~10 ms processor delay plus an additional 90 ms programmed delay) conditions by recording the input and output signals of the processor simultaneously (using an external sound card; Tascam US2 × 2) and measuring the input–output latency.

The target words consisted of meaningful monosyllabic consonant-vowel-consonant words such that the vowel was always a front vowel (/i/, /ɪ/, /ɛ/, or /æ/; e.g., bead, bit, pet, map). Because the purpose of the study was to compare the trial-to-trial variability of AWS and ANS, we selected monosyllabic words to reduce the likelihood of disfluency in the stuttering participants; thus, we could compare variability in fluent speech of AWS with the speech variability of ANS. The rationale for using a 100-ms delay was based on previous literature that has shown delays of around 100 ms (compared with longer delays) to be more effective in improving fluency in adults who stutter (Lincoln et al., 2006). Additionally, because our study’s target words were monosyllabic, which typically have durations between 200 and 300 ms (Haley et al., 2023; Lehiste, 1972), participants speaking under the DAF of 100 ms would still be producing the words when they heard the delayed feedback, likely maximizing DAF’s impact on their speech.

### Data analysis

We used the Audapter formant tracking module (in offline mode) to calculate formant frequencies for each production (Cai, 2015). Audapter is a software package that uses linear predictive coding (LPC) analysis and dynamic programming to calculate formant frequencies in both offline and real-time modes. For formant calculation, we used an LPC order of 17 for male participants and 15 for female participants. Blinded with regard to condition (NAF or DAF) and group (AWS or ANS), a member of our research team visually inspected the formant trajectories and listened to the production to exclude formant tracking errors and mispronunciations. Note that because (1) participants were alone while speaking, (2) the target words were monosyllabic, and (3) the delay in the DAF condition was relatively short, participants did not produce disfluent speech during the NAF or DAF conditions. Approximately 5% of all trials were excluded (due to errors in estimated formant trajectories or low audio quality), and there were no statistically significant differences between groups (*p* = 0.912) or conditions (*p* = 0.127) in terms of the number of excluded trials.

While inspecting the productions, we manually selected the onset and offset of the vowel for each production. We then used the vowel onset and offset times to extract formant trajectories of the vowel. Because we were interested in feedforward processes, we examined the first 50 ms of the vowels to avoid feedback-driven changes in speech output (Daliri, 2021; Merrikhi et al., 2025). We used the Mel-scaled formant values in this time window to calculate trial-to-trial variability as the primary dependent variable. We estimated the trial-to-trial variability by calculating the average of the first formant (F1) and the average of the second formant (F2) for each trial, subtracting the grand average F1 and F2 from these formant values for each trial, and then calculating the root mean square of the adjusted formant values in each vowel and condition. To more thoroughly examine the acoustic data, we also estimated the within-trial variability by calculating the standard deviations of F1 and F2 trajectories and then calculating the root mean square of the standard deviations for all trials in each vowel and condition. Because variability measures are bounded on one end (cannot become less than zero), we normalized their distribution using a logarithmic transformation prior to entering into the statistical analyses. DAF typically leads to increased vowel duration and speech intensity (For a review, see Yates, 1963). Thus, we also examined vowel duration and intensity to confirm the overall impact of DAF on participants’ speech output. Additionally, a previous study reported that AWS’ vowels are less distinct than those of ANS (Blomgren et al., 1998; Robb et al., 1998); therefore, we also measured the distance between vowels to examine whether the AWS’ vowels are less distinct than those of ANS. Overall, we calculated three additional variables as secondary measures: (1) the average vowel duration, (2) the average vowel intensity, and (3) the average distance between the four vowels in the F1-F2 coordinates.

We conducted all statistical analyses using the R language (version 4.3.1) (R Core Team, 2020) in the RStudio environment (version 2023.06.2) (R Core Team, 2020). For each of the dependent variables, we used the lme4 package (Bates et al., 2015; Kuznetsova et al., 2017) to fit a linear mixed-effect model to the data. We used vowels (/i/, /ɪ/, /ɛ/, and /æ/), conditions (NAF and DAF), and groups (AWS and ANS) as fixed factors and participants as a random intercept. We determined the statistical significance of the main effects and interactions using the lmerTest package (Kuznetsova et al., 2017). For statistically significant interactions, we conducted post-hoc analyses using the emmeans package (Lenth, 2019) and corrected the significance for multiple comparisons using the Tukey method.

## Results

### The impact of DAF on vowel characteristics

**Figure 1:**
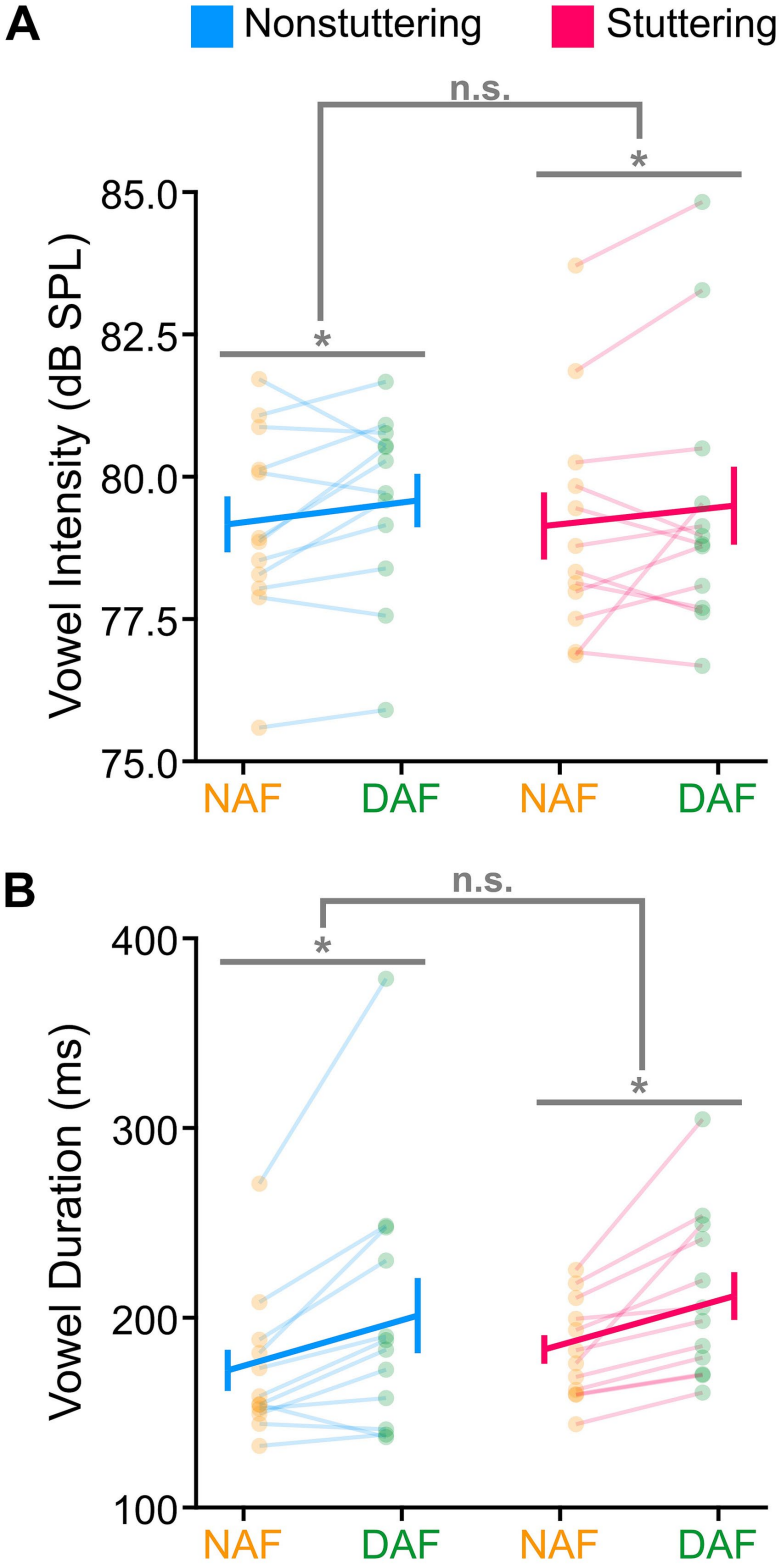
Generally, participants’ speech intensity **(A)** and vowel duration **(B)** were greater in the delayed auditory feedback (DAF) condition compared to the normal auditory feedback (NAF) condition. However, the stuttering and nonstuttering groups showed similar changes in their speech intensity and vowel durations in both conditions. Error bars correspond to the standard error of the mean. Asterisks correspond to significant differences (*p* < 0.05), and “n.s.” corresponds to nonsignificant differences.

**Figure 2:**
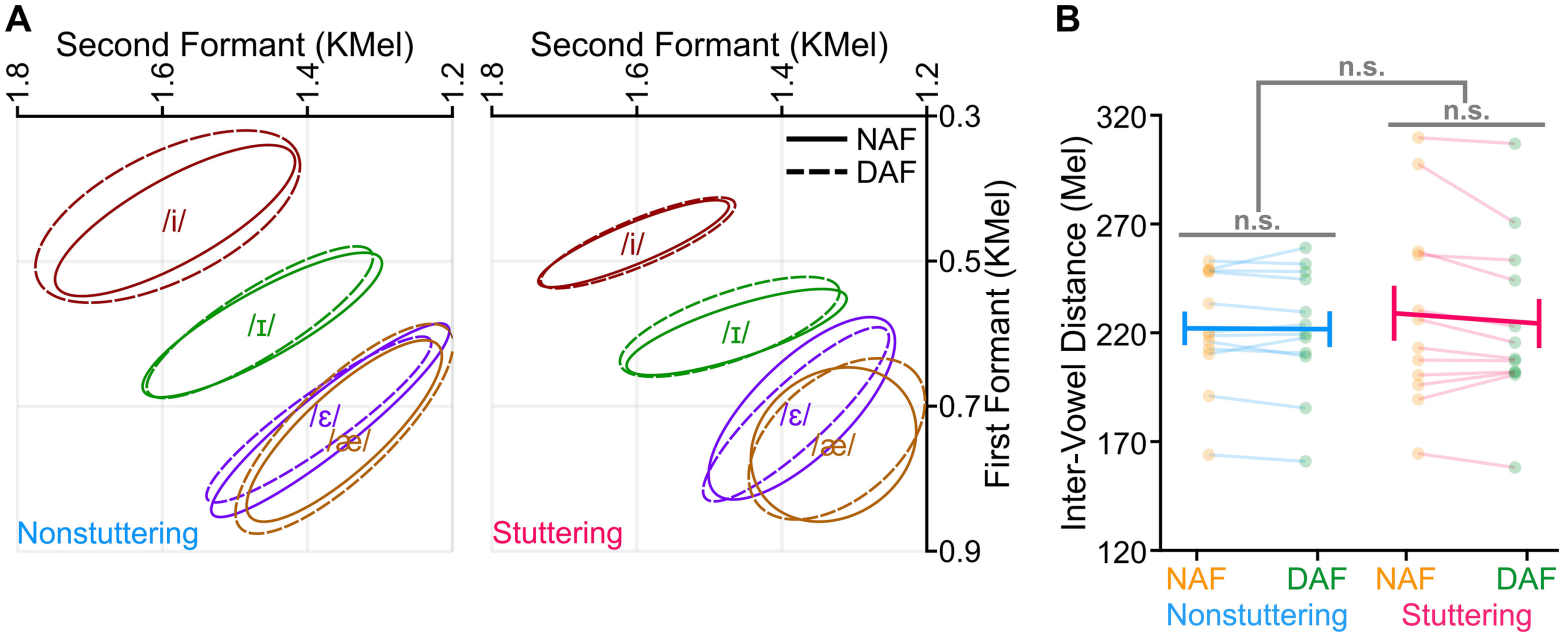
**(A)** Distribution of the produced vowels in F1-F2 coordinates for nonstuttering and stuttering participants in conditions with normal auditory feedback (NAF) or delayed auditory feedback (DAF). **(B)** The two groups showed similar average inter-vowel distances in both conditions. Error bars correspond to the standard error of the mean, and “n.s.” corresponds to nonsignificant differences.

In our initial analysis, we examined DAF’s impact on three vowel characteristics: intensity, duration, and inter-vowel distance. Analyses of vowel intensity revealed statistically significant main effects of vowel, *F* (3, 154) = 21.015, *p* < 0.001, and condition, *F* (1, 154) = 14.569, *p* < 0.001. These main effects indicated that (1) the intensity of /i/ was lower than /ɪ/, /ɛ/, and /æ/ (*p* < 0.001), and (2) intensity in the NAF condition was lower than in the DAF condition (Figure 1A). We did not find a statistically significant main effect of group (*p* = 0.939) or any statistically significant two-way or three-way interactions (*p* > 0.298 in all cases). Examining vowel duration, we found statistically significant main effects of vowel, *F* (3, 154) = 99.991, *p* < 0.001, and condition, *F* (1, 154) = 94.704, *p* < 0.001. These results indicated that (1) the duration of /æ/ was longer than that of other vowels, and (2) duration was longer in the DAF condition compared with the NAF condition (Figure 1B). The main effect of group (*p* = 0.561) and all interactions were not statistically significant (*p* > 0.365). Additionally, analyses of the average inter-vowel distance revealed no statistically significant condition effect (*p* = 0.124), group effect (*p* = 0.742), or group by condition interaction (*p* = 0.189). Figure 2A shows the distribution of the vowels in the F1-F2 coordinates for the two groups and in both conditions. The two groups’ average inter-vowel distance (averaged across all four vowels) was similar in both conditions (Figure 2B). Overall, these results indicated that there were no statistically significant differences between the AWS and ANS in terms of the duration and intensity of vowels or in terms of the distance between vowels in the F1-F2 coordinates in the NAF and DAF conditions.

### The impact of DAF on vowel production variability

**Figure 3:**
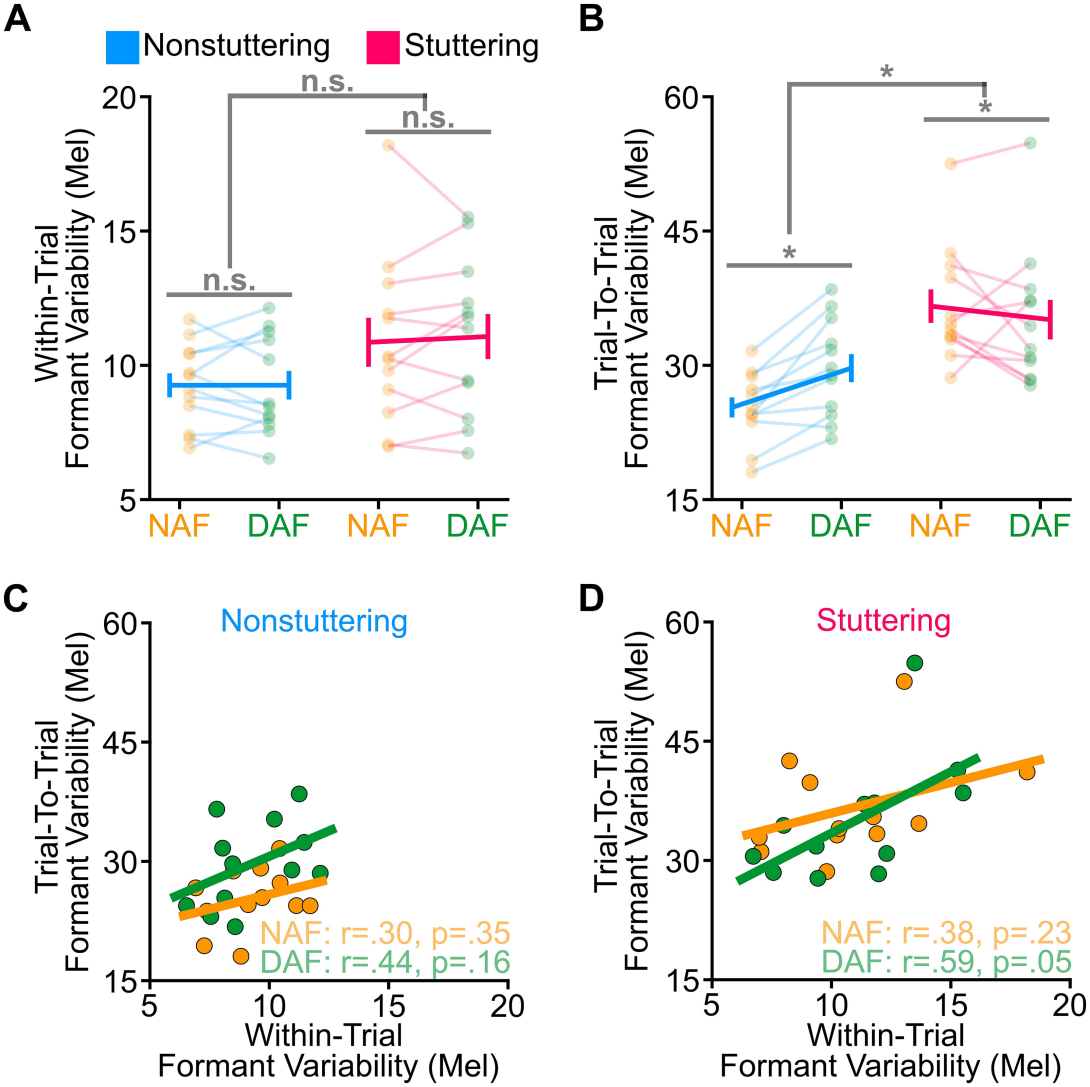
Stuttering and nonstuttering participants showed similar within-trial variability **(A)** but different trial-to-trial variability **(B)**. Our analysis of trial-to-trial variability revealed a significant group effect and a significant group-by-condition interaction, indicating higher variability among stuttering participants compared to nonstuttering participants, a decrease in variability among stuttering participants, but an increase in variability among nonstuttering participants when speaking with delayed auditory feedback (DAF) versus normal auditory feedback (NAF). Correlations between within-trial variability and trial-to-trial variability are shown separately for the nonstuttering group **(C)** and stuttering group **(D)**. Error bars correspond to the standard error of the mean. Asterisks correspond to significant differences (*p* < 0.05), and “n.s.” corresponds to nonsignificant differences.

This study had two primary goals: first, to examine whether stuttering affects trial-to-trial formant variability; and second, to examine the effect of DAF on this variability in the same population, given DAF’s potential to enhance feedforward processing. Examining trial-to-trial variability, we found statistically significant main effects of vowel, *F* (3, 154) = 25.785, *p* < 0.001, and group, *F* (1, 22) = 16.971, *p* < 0.001. We also found a statistically significant group by condition interaction, *F* (1,154) = 5.568, *p* = 0.019 (Figure 3B). However, we did not find a statistically significant condition effect (*p* = 0.188), vowel by group interaction (*p* = 0.222), vowel by condition interaction (*p* = 0.465), or vowel by group by condition interaction (*p* = 0.724). Post-hoc analyses indicated that trial-to-trial variability (1) was the lowest for /i/ as compared with other vowels (*p* < 0.001), (2) was generally higher among AWS relative to ANS (*p* < 0.001), (3) increased in ANS when speaking with DAF versus NAF (*p* = 0.036), and (4) decreased in AWS when speaking with DAF versus NAF (*p* < 0.001). In contrast, for within-trial variability (Figure 3A), we found only a statistically significant main effect of vowel, *F* (3,154) = 47.592, *p* < 0.001, with /æ/ having higher within-trial variability than the other vowels. Our analysis did not reveal statistically significant main effects of group (*p* = 0.122) and condition (*p* = 0.765) or two-way or three-way interactions (*p* > 0.387 in all cases). Similarly, we did not find significant relationships between within-trial variability and trial-to-trial variability for both groups in each of the conditions (Figures 3C,D). We also did not find a statistically significant relationship between stuttering severity measures (SSI score, stuttering frequency, and severity classification) with any of the variability measures (*p* > 0.304).

## Discussion

The goals of this study were to examine (1) whether the speech of adults who stutter is characterized by atypical trial-to-trial formant variability (as a behavioral measure of feedforward motor processes) in individuals who stutter, and (2) whether DAF leads to a reduction in trial-to-trial formant variability of stuttering individuals. Adults who stutter and matched adults who do not stutter produced front vowels in the context of monosyllabic consonant-vowel-consonant words when speaking under NAF and DAF conditions. We used trial-to-trial variability in the formant frequencies of the initial portion of their vowel as a behavioral correlate of feedforward processes. Our analyses showed that (1) relative to ANS, AWS generally had higher trial-to-trial variability, and (2) DAF decreased trial-to-trial variability of AWS, but it increased trial-to-trial variability of ANS.

Our findings of increased trial-to-trial formant variability in AWS align with our first hypothesis, suggesting that stuttering may be associated with inefficiencies in feedforward processes. Given that we calculated trial-to-trial variability using the first 50 ms of the vowel, the measured trial-to-trial changes in speech are primarily driven by feedforward processes (Kearney et al., 2020; Li et al., 2024; Merrikhi et al., 2025). During this early period of the vowel, the brain has not yet processed the auditory feedback related to the produced speech and has not started to correct potential inaccuracies in the production. Several previous studies have used the early period of vowels to isolate the contributions of the feedforward processes without the influence of feedback-driven corrections (Chao and Daliri, 2023; Guenther, 2016; Kearney et al., 2020; Merrikhi et al., 2025). Thus, the finding that AWS had higher trial-to-trial variability suggests less efficient feedforward processes, resulting in more variable speech output from trial to trial. Moreover, the finding that trial-to-trial variability in AWS is reduced under DAF is consistent with our second hypothesis, suggesting that altered auditory feedback may cause AWS to use a different motor planning strategy (such as allocating more neural resources to planning) (e.g., Lincoln et al., 2006). This adjusted strategy may compensate for inefficiencies in the feedforward processes, resulting in less variability. On the other hand, DAF interferes with the feedforward processes in ANS, thereby increasing their trial-to-trial variability.

Although the hypothesis of inefficient feedforward processes in AWS generally explains our findings, it does not specify the precise nature of the inefficiency. As a new hypothesis for further testing, we propose that movement variability is a byproduct of (1) less accurate motor commands and (2) responses to the resulting errors perceived in previous trials. The brain relies on feedforward and feedback control systems to produce accurate and fluent speech (e.g., Guenther, 2016). The feedforward control system prepares motor commands to achieve specific auditory targets. The feedback control system monitors the auditory outcomes of the motor commands (auditory feedback) to estimate potential errors in the produced speech (e.g., Houde and Nagarajan, 2011). The brain then uses the estimated errors to update future feedforward motor commands to reduce errors in subsequent productions. Therefore, if for any given production the brain selects motor commands from a wide distribution of possible options (e.g., a normal distribution with high variance), there will be significant trial-to-trial variability (Berret et al., 2021). Additionally, if the brain is too “sensitive” to errors in prior productions, it will make larger adjustments in its selection of feedforward motor commands for each trial, resulting in increased trial-to-trial variability. We should note that error sensitivity in this context refers to the weight the brain assigns to errors when computing the magnitude of its response to those errors (Daliri, 2021; Daliri and Dittman, 2019; Houde and Nagarajan, 2011; Merrikhi et al., 2025). Based on this proposal, our finding of higher trial-to-trial variability in AWS may suggest that AWS have (1) more variable motor commands and (2) higher sensitivity to small deviations in their speech, leading to excessive trial-to-trial changes in their motor commands (Shadmehr and Mussa-Ivaldi, 2012). Our findings of a decrease in trial-to-trial variability of AWS under DAF and an increase in variability of ANS may suggest that (1) AWS have lower sensitivity to the large errors induced by DAF, leading to smaller trial-to-trial changes in their motor commands and (2) ANS respond to the induced errors, leading to larger trial-to-trial changes in their motor commands in the DAF condition. Overall, one possible explanation, which needs to be tested in future studies, is that AWS might exhibit higher error sensitivity for small errors (responding more strongly to them) and lower sensitivity for large errors (discounting them more drastically) compared to ANS. Based on this interpretation, AWS would implement larger adjustments in response to small deviations in their speech when speaking under NAF—leading to higher trial-to-trial variability—whereas ANS would implement smaller adjustments when speaking under NAF—leading to smaller trial-to-trial variability. Given that DAF induces large errors, AWS would discount the errors and, thus, make smaller adjustments in their speech (i.e., reducing trial-to-trial variability in the DAF condition relative to the NAF condition). In contrast, ANS would respond more to the large error induced by DAF and thus make more adjustments in their speech (i.e., increasing trial-to-trial variability in the DAF condition relative to the NAF condition). This interpretation can also explain AWS’ reduced (or their lack of response) adaptive and corrective responses to auditory feedback perturbations, given that most auditory feedback perturbations induce large errors (Cai et al., 2012; Daliri et al., 2017; Daliri and Max, 2018; Frankford et al., 2022, 2023; Kim et al., 2020; Loucks et al., 2012).

Our suggestion of AWS’ higher sensitivity to small errors is in general agreement with contemporary views of malfunctioning processes in stuttering. A commonly reported hypothesis is that stuttering is associated with overreliance on auditory feedback (Civier et al., 2010; Max, 2004; Max et al., 2004). This hypothesis suggests that stuttering stems from malfunctioning processes that lead stuttering individuals to overreliance on auditory feedback for speech production. Overreliance on auditory feedback may make individuals more sensitive to errors reported by the auditory feedback (especially small errors), increasing trial-to-trial variability. Another commonly reported hypothesis in the literature is the vicious cycle hypothesis (Vasic and Wijnen, 2005). This hypothesis suggests that stuttering individuals have overactive speech-monitoring processes such that even minor and typical deviations in speech plans are considered errors that need to be corrected. Again, AWS’ higher sensitivity to small errors is in agreement with this hypothesis, with the exception that we speculate that AWS discount large errors and do not adjust their speech in response to larger errors. Overall, while our interpretation of higher trial-to-trial variability is in line with previous views on stuttering, it extends those views such that it limits stuttering individuals’ overactive error reliance to small errors.

A secondary finding of this study was the impact of the DAF on vowel characteristics, which were similar across the two groups. Our results showed that vowel intensity and duration generally increased in the DAF condition. These results are consistent with previous reports on the impact of DAF on speech output (Chon et al., 2013; Yates, 1963). We also found that the intensity of /i/ was lower than that of other vowels, and the duration of /æ/ was the longest. These findings are consistent with previous reports (e.g., Hillenbrand et al., 1995). Studies have often explained these patterns in vowel characteristics through articulatory constraints required for producing vowels (Perkell, 2012; Perkell and Zandipour, 2002). Additionally, we found that trial-to-trial variability was the lowest for /i/ compared to other vowels, consistent with previous reports (e.g., Hillenbrand et al., 1995). Articulatory configurations of /i/ may explain this lower variability, as it requires a smaller constriction in the overall cavity, resulting in a possibly stronger somatosensory target (Ghosh et al., 2010; Guenther, 2016; Perkell, 2012). Examining within-trial formant variability did not reveal between-group or between-condition differences; however, the within-trial variability of /æ/ was highest among all vowels for both groups. The increased within-trial variability observed for the vowel /æ/ may stem from its demanding articulatory configuration, which requires a larger mouth opening, involving a lowered jaw and tongue. Since the preceding consonant often involves oral cavity closures, the initial part of /æ/ necessitates larger, more rapid articulatory movements to reach the target position (i.e., from a closure to a large opening in the oral cavity). This articulatory constraint has also been linked to a reduced reliance on somatosensory feedback (Guenther, 2016; Perkell, 2012), which may further contribute to the increased variability during vowel production. While the absence of a between-group difference in within-trial variability might initially suggest intact feedback control mechanisms in AWS, this interpretation requires caution. Our design, which restricted the within-trial variability analysis to the vowel’s initial 50 ms, inherently minimized the impact of real-time feedback adjustments. Thus, the variability we measured more accurately reflects articulatory precision during the early, ballistic phase of vowel production, rather than the full operation of feedback loops. We should also note that the lack of between-group difference is in contrast with the results of a previous study that reported increased fluctuations in the second formant of AWS during consonant-vowel transitions (Robb et al., 1998); however, there are many important methodological differences between the current study and the previous study that may contribute to the apparent disparity between findings of the two studies. For example, our measure of within-trial variability is based on the first and second formants, whereas the previous study only focused on the second formant. Overall, our secondary findings indicated that the vowel characteristics were similar between AWS and ANS, and, as expected, the DAF influenced the production of both groups.

Our study also has several limitations that need to be considered when interpreting the findings. First, the lack of correlation between trial-to-trial variability and stuttering severity, coupled with the adult participant sample, limits their generalizability. Thus, future studies need to determine whether the increased trial-to-trial variability in AWS is a compensatory mechanism that they develop to cope with stuttering (e.g., Chon et al., 2021). Second, our study’s focus on monosyllabic words and a single-word reading task limits the generalizability of our findings to the complexities of real-world conversation. To more accurately assess the contribution of inefficient feedforward processes to increased trial-to-trial variability in stuttering, future research should investigate trial-to-trial variability within connected speech (such as sentences and phrases) and natural conversational contexts. Such paradigms would also allow for the examination of trial-to-trial variability in contexts where disfluency is more likely to occur in individuals who stutter. Future studies should also specifically include individuals who stutter with diverse severity levels and across their lifespan and explore the impact of DAF on trial-to-trial variability. Third, our study’s design, which aimed to isolate the contribution of feedforward processes, restricts the interpretation of our findings solely to the feedforward control system. However, stuttering may also be associated with deficiencies or inefficiencies in other sensorimotor processes, including the feedback control system. To expand upon this, future research can employ altered auditory feedback paradigms to explore potential relationships between adaptive and corrective responses to perturbations and trial-to-trial variability. Finally, the limited sample size in our study, particularly in conjunction with its complex factorial design (group × condition × vowel), constrained the statistical power and thus the generalizability of our findings. Therefore, replicating these findings with larger and more diverse participant cohorts is crucial. Overall, our findings have several limitations, which may restrict their interpretation and generalizability, and should be considered as a foundation for future studies.

In summary, we examined the variability of vowel production by examining the variability of formant frequencies. Stuttering and nonstuttering adults produced vowels in the context of monosyllabic consonant-vowel-consonant words when speaking under NAF or DAF. We measured within-trial and trial-to-trial formant variability for each participant. Our analyses showed that (1) relative to ANS, AWS generally had higher trial-to-trial variability, and (2) DAF decreased trial-to-trial variability of AWS, but it increased trial-to-trial variability of ANS. These findings indicate that stuttering adults generated more variable feedforward motor commands than nonstuttering adults when speaking with NAF, but that speaking with DAF decreased variability in the stuttering group, whereas it increased variability in the nonstuttering group. One possible interpretation, which future studies will need to test, is that stuttering modulates the sensorimotor system’s processing of auditory errors, specifically influencing how significantly these errors are weighted when the system determines its responses. Finally, while many studies have reported abnormalities in several distinct brain networks of stuttering individuals (Chang and Guenther, 2020; Etchell et al., 2018; Neef and Chang, 2024), the neural basis of our behavioral findings remains to be determined. Specifically, future studies need to determine whether the increased trial-to-trial variability (or the differential effect of DAF on the variability) in stuttering individuals results from suboptimal activity in brain networks such as the (pre-)motor-auditory and cortico-basal ganglia-thalamocortical networks.

## Data Availability

The datasets presented in this article are not readily available because the dataset collected for the current study is available upon reasonable request from the first author (A. Daliri). Requests to access the datasets should be directed to Ayoub Daliri, Ayoub.Daliri@asu.edu.
